# Exploring the Thermodynamic Uncertainty Constant: Insights from a Quasi-Ideal Nano-Gas Model

**DOI:** 10.3390/e26121011

**Published:** 2024-11-23

**Authors:** Giorgio Sonnino

**Affiliations:** Department of Physics, Université Libre de Bruxelles (U.L.B.), Campus de la Plaine C.P. 224, Bvd du Triomphe, 1050 Brussels, Belgium; giorgio.sonnino@ulb.be

**Keywords:** entropy, mesoscopic scale, canonical commutation rules, thermodynamics of irreversible processes, Prigogine and Onsager’s theory, complex systems

## Abstract

In previous work, we investigated thermodynamic processes in systems at the mesoscopic level where traditional thermodynamic descriptions (macroscopic or microscopic) may not be fully adequate. The key result is that entropy in such systems does not change continuously, as in macroscopic systems, but rather in discrete steps characterized by the quantization constant β. This quantization reflects the underlying discrete nature of the collision process in low-dimensional systems and the essential role played by thermodynamic fluctuations at this scale. Thermodynamic variables conjugate to the forces, along with Glansdorff–Prigogine’s dissipative variable can be discretized, enabling a mesoscopic-scale formulation of canonical commutation rules (CCRs). In this framework, measurements correspond to determining the eigenvalues of operators associated with key thermodynamic quantities. This work investigates the quantization parameter β in the CCRs using a nano-gas model analyzed through classical statistical physics. Our findings suggest that β is not an unknown fundamental constant. Instead, it emerges as the minimum achievable value derived from optimizing the uncertainty relation within the framework of our model. The expression for β is determined in terms of the ratio χ, which provides a dimensionless number that reflects the relative scales of volume and mass between entities at the Bohr (atomic level) and the molecular scales. This latter parameter quantifies the relative influence of quantum effects versus classical dynamics in a given scattering process.

## 1. Introduction

Mesoscopic structures are systems or materials that exist on a scale between microscopic (atomic or molecular) and macroscopic (bulk) levels and constitute the building blocks of nanotechnology and modern electronics. Devices such as nanoscale transistors and single-electron transistors rely on the unique properties of materials at the mesoscopic scale to achieve high performance and efficiency [1,2,3]. Mesoscopic materials, like graphene and other 2D materials, exhibit novel electronic, thermal, and mechanical properties that are exploited in advanced material design for stronger, lighter, and more efficient materials [4,5,6]. Furthermore, mesoscopic systems are highly sensitive to their environment, making them excellent candidates for use in precision sensors and measurement devices, such as nanoscale thermometers, biosensors, and environmental detectors [7,8,9]. These systems go beyond the traditional dichotomy of classical versus quantum physics. Ref. [10], the authors addressed the need for a deeper understanding of dynamics and thermodynamics at the mesoscopic level by pointing out that traditional quantum mechanics and classical thermodynamics do not fully capture the essence of mesoscopic systems. It has become increasingly clear that mesoscopic systems display behaviors that operate in an intermediate regime governed by unique principles distinct from classical and quantum domains. In mesoscopic systems, irreversibility and the discrete nature of the collision process in low-dimensional systems play an essential role, particularly in non-equilibrium thermodynamics. Additionally, these systems are small enough that they experience significant fluctuations, but they are not so small that quantum mechanical uncertainty alone dominates. Building on one of the primary research aims of the Brussels School of Thermodynamics, inspired by I. Prigogine, it is crucial to study the physics and technology of nanomaterials to uncover fundamental principles and new laws that govern systems at the mesoscopic scale. This research bridges the gap between macroscopic and microscopic behavior, enhancing our understanding of complex phenomena. In [10], it was shown that canonical commutation rules (CCRs) can be proposed for pairs of variables such as (time, entropy production) and (thermodynamic variable, conjugate thermodynamic force). These CCRs, together with the second quantization approach, provide the essential physical and mathematical framework to address stochastic effects at the mesoscopic scale. The concept of entropy production as an operator with quantized eigenvalues introduces a groundbreaking perspective for mesoscopic systems: while traditional thermodynamics assumes continuous entropy changes, at the mesoscopic scale, entropy may be produced in discrete units, much like energy quantization in quantum systems. This reflects the intermediate nature of these systems, where neither classical nor quantum mechanics alone suffices. In classical physics, time is usually treated as a parameter, but in mesoscopic systems, the interaction of time with thermodynamic quantities, such as entropy, may necessitate treating time itself as an operator. This is a profound shift, as it introduces a more dynamic and interactive view of time in thermodynamic processes, which could capture mesoscopic systems’ complexity better than classical descriptions. These new mesoscopic laws have the potential to influence how we design technologies at the nanoscale, particularly in fields such as thermodynamics of small systems, nanoelectronics, and materials science, where controlling entropy production and time-dependent processes is crucial. Motivated by the goals outlined above and supported by recent experimental findings in [11], our study in [10] examines thermodynamic processes within small systems operating in Onsager’s region. Following the general formalism introduced in [10], canonically conjugate variables obey the CCRs, where measurements involve determining the eigenvalues of operators associated with thermodynamic quantities. The focus of that work was to explore the implications of these postulates. In this study, we aim to understand the nature of the quantization parameter β, which appears in the CCRs. Specifically, two open questions drive this investigation: Is β a new fundamental constant? And does it represent a minimum limit within the CCRs? To address these questions, we begin with a simple assumption: that β can be derived from a straightforward, heuristic model for nano-gas and analyzed using classical statistical physics. Our model indicates a negative answer to the first question but an affirmative answer to the second. Additionally, the theoretical value of β aligns closely with the experimental result found in [11].

This manuscript is structured as follows. Section 2 introduces background information to establish the necessary vocabulary. Section 3 provides a brief overview of the formalism used to quantize entropy production strength at the mesoscopic scale, offering readers a foundation to understand this work independently of [10]. For a more detailed exploration of the Onsager region, including the discretization of key thermodynamic quantities—such as total entropy production, thermodynamic variables conjugate to their forces, and the Glansdorff–Prigogine dissipative variable—refer to [10]. In Section 4, we present the derivation of the β constant in the canonical commutation rules (CCRs) and examine its physical origin. Finally, Section 6 and Section 7 provide perspectives on future directions and concluding remarks, respectively.

## 2. Background

To set the foundation for our discussion, we start with two brief subsections: the first outlines key aspects of Prigogine’s formulation of thermodynamic processes within the Onsager regime, and the second reviews the definition of the space in which calculations are performed. Importantly, the mathematical framework relies on the Fourier transform, which typically assumes periodic boundary conditions or an infinitely large interval. Here, however, the Fourier transform is applied within the space of thermodynamic forces rather than physical space.

### 2.1. Prigogine’s Second Law of Thermodynamics and Onsager’s Region

Consider a system characterized by *n degrees of advancement* $\xi_{1},\ldots ,\xi_{n}$. It is worth recalling that this terminology is due to Th. De Donder [12,13,14,15,16]. According to Prigogine’s book [17], *degrees of advancement* in nonequilibrium thermodynamics refers to the progressive stages of theoretical and experimental understanding that extend thermodynamic principles to systems driven out of equilibrium, emphasizing entropy production, energy fluxes, and time-dependent processes across various scales. For instance, in chemical reactions, it is often useful to consider the *k* mole numbers $n_{1},\ldots ,n_{k}$. We then have $dn_{i}=\nu_{i}d\xi$ with $\nu_{i}$ denoting the stoichiometric coefficients. dξ is called *degree of advancement* or, in the specific case of chemical reactions, *extent of the reaction*. The deviations of $\xi_{i}$ from their equilibrium values $\xi_{\mu }^{eq.}$, which correspond to the degrees of advancement in a state of local equilibrium, are denoted by ${\bar{\alpha }}_{\mu }$, defined as ${\bar{\alpha }}_{\mu }\equiv \xi_{\mu }-\xi_{i}^{eq.}$. Thus, ${\bar{\alpha }}_{\mu }$ can represent fluctuations in various thermodynamic quantities such as pressure, the extent of the reaction, etc. The role of temperature fluctuations is extensively discussed in several works e.g., in [18,19,20]. Following the principles of thermodynamics, we can define a state function *S*, representing the entropy of the system, which possesses certain properties. The entropy variation resulting from these fluctuations is given by by [17,21,22]

(1) $$\Delta S=\Delta_{e}S+\Delta_{I}S$$

where

(2) $$\begin{array}{cc} \; & \Delta S=\int_{\xi^{eq.}}^{\xi }dS=S\left(\xi \right)-S\left(\xi^{eq.}\right)\\ \; & \Delta_{e}S=\int_{\xi^{eq.}}^{\xi }d_{e}S\;;\;\Delta_{I}S=\int_{\xi^{eq.}}^{\xi }d_{I}S \end{array}$$

$\delta S_{e}$ represents the entropy added to the system by its surroundings, while $\Delta_{I}S$ indicates the entropy generated within the system. According to the second law of thermodynamics, $\Delta_{I}S$ must equal zero for reversible (or equilibrium) transformations, and it must be positive for irreversible transformations, which means $\Delta_{I}S\geq 0$. In contrast, the entropy supplied, $\Delta_{e}S$, can be positive, zero, or negative, depending on how the system interacts with its surroundings. Additionally, denoting the volume occupied by the system with *V*, we have [23];

(3) $$\begin{array}{cc} \; & \bar{\sigma }=\frac{\delta }{\delta V}\left(\frac{d\Delta_{I}S}{dt}\right)=\frac{d}{dt}\left(\frac{\delta \Delta_{I}S}{\delta V}\right)=\frac{\partial \Delta_{I}s}{\partial {\bar{\alpha }}_{\mu }}\frac{d{\bar{\alpha }}_{\mu }}{dt}=x^{\mu }j_{\mu }\\ \; & \mathrm{with}\;j_{\mu }\equiv \frac{d{\bar{\alpha }}_{\mu }}{dt} \end{array}$$

with *s* denoting the entropy density (s=δS/δV). Here, $j_{\mu }$ represents the *thermodynamic fluxes* associated with the thermodynamic force $x^{\mu }$, and δV denotes an infinitesimal spatial volume element occupied by the system. In Equation (3), we have employed the Einstein convention of repeated indices. This convention will also be utilized throughout the remainder of this manuscript unless stated otherwise. To work with the entropy production strength, which has the dimension of [Energy]/([Temperature] × [time]), we define the thermodynamic forces, thermodynamic fluxes, and thermodynamic variables as follows:

(4) $$X^{\mu }\left(r,t\right)\equiv \;\sqrt{V}x^{\mu }\;;\;J_{\mu }\left(r,t\right)\equiv \;\sqrt{V}j_{\mu }\;;\;\alpha_{\mu }\left(r,t\right)\;\equiv \;\sqrt{V}{\bar{\alpha }}_{\mu }$$

Here, (r,t) represents the space–time coordinates. Equation (3) connects the entropy production strength to the thermodynamic forces and their corresponding conjugate fluxes. To express the entropy production strength solely in terms of the thermodynamic forces, it is essential to establish a relationship between the dissipative fluxes and the thermodynamic forces that generate them. These relationships are known as *transport flux–force relations*. In thermodynamic systems within Onsager’s region, the most commonly employed transport relations are [24,25]:

(5) $$J_{\mu }=L_{\mu \nu }X^{\nu }$$

where $L_{\mu \nu }=L_{\nu \mu }$. Quantity $L_{\mu \nu }$ is referred to as *Onsager’s Matrix*, with its entries representing the transport coefficients that are independent of the thermodynamic forces. Notice that in his work [17], Prigogine demonstrated the validity of Onsager’s reciprocity relation only for small spontaneous fluctuations around thermodynamic equilibrium. In contrast, [10] presented a more general result, showing that, under the assumption of microscopic reversibility, the formalism of the canonical commutation relations ensures the validity of Onsager’s reciprocity relations throughout the entire linear region of thermodynamics, not just in the vicinity of the ground state. It is important to express the transport coefficients in a dimensionless form for calculation purposes. In Onsager’s region, the local entropy production strength can be expressed in terms of these transport coefficients as follows:

(6) $$\sigma =L_{\mu \nu }X^{\mu }X^{\nu }=VL_{\mu \nu }x^{\mu }x^{\nu }=V\bar{\sigma }$$

In such a way, σ has the following dimension:

[Energy]/([Temperature]×[time]) while

$\bar{\sigma }$ has a dimension of [Energy]/([Temperature]×[time]×[Volume]) (as we wish).

### 2.2. Defining the Metric and Affine Connection for Calculations in the Thermodynamic Force Space

**Figure 1:**
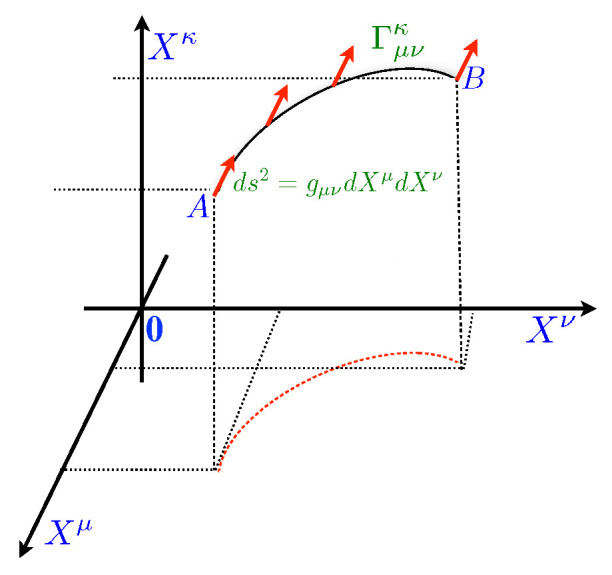
*The Thermodynamic Space*. The space is spanned by the thermodynamic forces, the metric tensor is identified with the symmetric piece of the transport coefficients, and the expression of the affine connection is determined by the *Universal Criterion of Evolution*.

To advance with the formalism, it is essential to define the space in which we can conduct our calculations. This requires specifying two key quantities: the *metric tensor* and the *affine connection* [26,27,28,29,30,31]. Both the metric tensor and the affine connection are derived from physical principles. The metric tensor corresponds to the symmetric component of the transport coefficients, while the expression for the affine connection is established by enforcing the Glansdorff–Prigogine *Universal Criterion of Evolution*. Specifically,

**(a)**  A metric tensor is a central object in the theory; it describes the local geometry of space. The metric tensor is a dimensionless symmetric tensor used to raise and lower the indicative tensors and generate the connections used to determine the field equations, that have to be satisfied by the metric tensor and to construct the Riemann curvature tensor.
**(b)**  The curvature of a space can be identified by taking a vector at some point and transporting it parallel along a curve in space-time. An affine connection is a rule that describes how to legitimately move a vector along a curve on the variety without changing its direction. We adopt the following definitions: *the space of the thermodynamic forces* (or, simply, *the thermodynamic space*) is the space spanned by the thermodynamic forces. The metric tensor and the affine connection are determined by physics. More specifically, the metric tensor is identified with the symmetric piece $g_{\mu \nu }$ of the transport coefficients, and the expression of the affine connection $\Gamma_{\mu \nu }^{\kappa }$ is determined by imposing the validity of the Glansdorff-Prigogine *Universal Criterion of Evolution* [18,28,31,32]. Note that, for the second law of thermodynamics, the square (infinitesimal) distance $ds^{2}=ds\cdot ds$ is always a non-negative quantity—see Figure 1.

According to the second law of thermodynamics, the squared distance between two infinitely close points in the space of thermodynamic forces is always non-negative. In this work, we limit ourselves to dealing with systems in the Onsager region. So, the affine connection vanishes in this region. Furthermore, in the thermodynamic force space, the total entropy production $\sigma_{T}$ in Onsager’s region is expressed as follows:

(7) $$\sigma_{T}\left(t\right)=\frac{1}{V}\int_{V}L_{\mu \nu }X^{\mu }X^{\nu }\sqrt{L}\;d^{n}X$$

Here, *L* represents the determinant of Onsager’s matrix.

## 3. Quantization of the Entropy Production Strength

### 3.1. A Heuristic Approach

One of the primary goals of the Brussels School of Thermodynamics, established by Th’ephile De Donder and Ilya Prigogine, was to explore systems at the mesoscopic scale to uncover the fundamental laws that govern them. We begin our analysis with the following (heuristic) observation: a *quasi-localized* disturbance in entropy production can be represented through a linear combination of modes with similar mode numbers. Thus, when the system is influenced by *n* independent thermodynamic forces, a *local disturbance* in the strength of entropy production can be expressed as a superposition of plane waves characterized by a generic wave number K that satisfies the periodicity conditions:

(8) $$\sigma \left(X,t\right)=\frac{1}{{\left(2\pi \right)}^{n}}\int_{-\infty }^{+\infty }\left(\sigma_{K}e^{i(K\cdot X-\omega_{K}t)}+\sigma_{K}^{★}e^{-i(K\cdot X-\omega_{K}t)}\right)dK$$

Notice that the modes exist in the space of thermodynamic forces rather than in conventional spatial dimensions. As is well known, Fourier’s theorem stipulates:

(9) $$\Delta t\Delta \omega \geq 1\;;\;\Delta K_{\mu }\Delta X^{\mu }\geq 1\;\left(\mathrm{no}\;\mathrm{summation}\;\mathrm{convention}\;\mathrm{on}\;\mu ;\;\mu =1,\ldots ,n\right)$$

Recent experimental findings suggest a heuristic notion that, on a mesoscopic scale within the space of thermodynamic forces, the strength of entropy production is proportional to the frequency [11]. In [33] the authors investigate entropy production in nonequilibrium steady states (NESS) of mesoscopic quantum systems, analyzing its dependence on system parameters, including frequencies and fluctuations. Ref. [34], the authors focused on bounding entropy production rates in stochastic systems using waiting time distributions, providing experimentally accessible methods to measure nonequilibrium dynamics. Additionally, the thermodynamic variable $\alpha_{\mu }$, which is conjugate to the thermodynamic forces $X^{\mu }$, is proportional to the wave vector $K_{\mu }$, i.e.,

(10) σ=kBω;αμ,K=kBKμwithkB≡βkB

Here, kB denotes Boltzmann’s constant $k_{B}$ times a pure number, say β, undetermined at this stage. A combination of Equation (9) with Equation (10) yields

(11) ΔtΔσ≥kBΔαμΔXμ≥kB∀μ(nosummationconventiononμ;μ=1,…,n)

These inequalities impose a fundamental limit on the precision with which certain pairs of physical quantities—such as the entropy production rate and time or a thermodynamic force and its associated thermodynamic variable—can be predicted from initial conditions. In other words, it is impossible to determine both the entropy production rate of a system and time with precision at the same time; as our knowledge of the system’s entropy production becomes more accurate, our knowledge of time becomes less accurate, and vice versa. Likewise, there exists an uncertainty relationship between a thermodynamic force and its corresponding thermodynamic variable. The pairs of variables (t,σ) and $\left(\alpha_{\mu },X^{\mu }\right)$ can be referred to as *canonically conjugate*, drawing an analogy to the terminology used in quantum mechanics.

### 3.2. The Formalism of Second Quantization and the Thermodynamic Commutation Rules

We now face two main challenges: (i) the need to formalize the heuristic ideas presented in the previous subsection with rigorous mathematical structure, and (ii) the complexity of handling a system of entities with infinite degrees of freedom, as these entities are constantly produced and absorbed. The ideal mathematical framework for addressing these challenges is provided by the second quantization (SQ) formalism, commonly used in quantum field theory. The second quantization offers a systematic approach for managing large assemblies of identical entities [35]. This formalism involves the introduction of canonical commutation rules (CCRs), whereby physical quantities are ‘promoted’ to operator status. Notably, the use of CCRs is not limited to quantum mechanics; they are essential for managing vast numbers of identical entities that can be created and annihilated. Consequently, the SQ framework allows us to elevate the variables σ and *t* along with $X^{\mu }$ and $\alpha_{\mu }$ to the level of operators acting within the space of the states of the system and to impose specific constraints on these operators to achieve the desired product behaviors (see also [10]). At the mesoscopic scale, we express this as follows:

(12) [t,σ]=ikB2[αμ,K,XK′ν]=ikB2δμνδKK′withkB=βkB

Here, […] denotes the commutator of two operators, defined as [A,B]=AB−BA, while $\delta_{\mu \nu }$ represents the Kronecker delta. In [11], recent experiments measuring entropy production in non-equilibrium systems are reported. To our knowledge, this work presents the most accurate value for the lowest limit of this constant available in the literature. The authors provide several experimental traces illustrating the tip positions of various mechano-sensory hair bundles over time. They estimated the local irreversibility measure based on single 30-s recordings of the observed oscillations with a sampling rate of ω = 2.5 kHz (for more information, see the forthcoming Section 5.4). From these experiments, we can derive an approximate numerical value for β, finding $\beta ∼1.2\times 10^{-8}$, which implies kB∼1.6×10−31J/K. In the upcoming section, we shall provide an approximate expression of the constant β for a quasi-ideal gas, revealing that in this context, β does not appear to be a new fundamental constant. Instead, it emerges as the minimum achievable value derived from optimizing the uncertainty relation within the framework of our model.

### 3.3. Discrete Representation of Total Entropy Production Strength in the Onsager Regime

The expression for the *total* entropy production strength is

(13) $$\sigma_{T}\left(t\right)=\frac{1}{V}\;\;\;\int_{V}\;\;\sigma \left(X,t\right)\;\sqrt{L}d^{n}X\;\;=\;\;\frac{1}{V}\;\;\;\int_{V}\;\;L_{\mu \nu }X^{\mu }X^{\nu }\;\sqrt{L}d^{n}X$$

where *local* entropy production strength is split into two contributions

(14) $$\sigma \left(X,t\right)=L_{\mu \nu }X^{\mu }X^{\nu }=\frac{1}{2}L_{\mu \nu }X^{\mu }X^{\nu }+\frac{1}{2}L^{\mu \nu }J_{\mu }J_{\nu }$$

Since $L_{\mu \nu }$ is a positive definite matrix, there exists a matrix $A_{\nu }^{\mu }$ such that

(15) $$\;\;\;{X^{\prime }}^{\lambda }=A_{\kappa }^{\lambda }X^{\kappa }\;\mathrm{with}\;A_{\nu }^{\mu }\;\mathrm{such}\;\mathrm{that}\;A_{\lambda }^{\alpha }L^{\lambda \kappa }A_{\kappa }^{\beta }=I^{\alpha \beta }$$

Here, $I^{\alpha \beta }$ denotes the Identity matrix. After transformation, we find $\sqrt{L}\to 1$ and

(16) $$\sigma_{T}\left(t\right)=\frac{1}{V^{\prime }}\int_{V^{\prime }}\sigma \left(X^{\prime },t\right)d^{n}X^{\prime }\;\mathrm{with}\;\sigma \left(X^{\prime },t\right)=\frac{1}{2}\sum_{\mu =1}^{n}\left(|{X^{\prime }}^{\mu }{|}^{2}+|J_{\mu }^{\prime }{|}^{2}\right)$$

In the space of the thermodynamic forces, the Fourier series expansion of the thermodynamic fluxes within a finite volume *V* is given by

(17) $$\;\;\;J_{\mu }^{\prime }\left(X^{\prime },t\right)\;=\;\;\sum_{K}\left(J_{\mu ,K}\left(t\right)e^{i(K\cdot X^{\prime })}\;\;+\;\;J_{\mu ,K}^{★}\left(t\right)e^{-i(K\cdot X^{\prime })}\right)$$

where

(18) $$\;\;\;J_{\mu ,K}\left(t\right)\;=\;i\omega_{K}\alpha_{\mu ,K}\left(t\right)\;\mathrm{and}\;J_{\mu ,K}^{★}\left(t\right)\;=\;-i\omega_{K}\alpha_{\mu ,K}^{★}\left(t\right)$$

Substituting Equation (17) and the expression for $X_{K}^{\mu }\left(t\right)$ (in this case, $X_{K}^{\mu }={X^{\mu }}_{\;\;\;\;-K^{\prime }}^{★}$) into Equation (16) yields

(19) $$\sigma_{T}\left(t\right)=2\sum_{\mu =1}^{n}\sum_{K}\left(|X_{K}^{\mu }\left(t\right){|}^{2}+|J_{\mu ,K}\left(t\right){|}^{2}\right)=2\sum_{\mu =1}^{n}\sum_{K}\left(|X_{K}^{\mu }\left(t\right){|}^{2}+\omega_{K}^{2}|\alpha_{\mu ,K}\left(t\right){|}^{2}\right)$$

where the orthogonality relation

(20) $$\frac{1}{V}\int_{V}e^{iK\cdot X}e^{-iK^{\prime }\cdot X}d^{n}X=\delta_{KK^{\prime }}$$

has been taken into account. This expression is identical to the Hamiltonian of a harmonic oscillator if we set the value of the mass=4, and we identify the following terms: *position* $\to \alpha_{\mu ,K}$, *momemtum* $\to 2X_{K}^{\mu }$, and *frequency* $\to \omega_{K}$ (see, for example, [36]). So, we first define two new dimensionless operators ${\tilde{X}}_{K}^{\mu }$ and ${\tilde{\alpha }}_{\mu ,K}$, as follows:

(21) X˜Kμ=2kBωKXKμ;α˜μ,K=2ωKkBαμ,K

In terms of these new variables, the expression for $\sigma_{T}\left(t\right)$ reads

(22) σT(t)=∑μ=1n∑KkBωK∣X˜Kμ∣2+∣α˜μ,K∣2

As for the case of the harmonic oscillator, we have to assume the validity of the following *canonical commutation rules* (CCRs):

(23) [α˜μ,K,X˜K′ν]=iδμνδKK′⇒[αμ,K,XK′ν]=ikB2δμνδKK′

The two operators of *creation* “$a_{K}^{(\mu )+}$” and *destruction* “$a_{K}^{\left(\mu \right)}$” can be introduced and defined as usual (see, for example, [37]):

(24) $$\begin{array}{cc} \; & a_{K}^{\left(\mu \right)}\;=\frac{1}{\sqrt{2}}\left({\tilde{\alpha }}_{\mu ,K}+i{\tilde{X}}_{K}^{\mu }\right);\;a_{K}^{(\mu )+}\;\;\;=\frac{1}{\sqrt{2}}\left({\tilde{\alpha }}_{\mu ,K}-i{\tilde{X}}_{K}^{\mu }\right)\\ \; & \left[a_{K}^{\left(\mu \right)},a_{K^{\prime }}^{(\mu^{\prime })+}\right]=\delta_{\mu \mu^{\prime }}\delta_{KK^{\prime }} \end{array}$$

In line with our expectation (see Section 3.1), we finally obtain the discretization of the total entropy production strength in Onsager’s region

(25) σT(t)=∑μ=1n∑KkBωKnK(μ)+12

where we have introduced the *number operator* $n_{K}^{\left(\mu \right)}\equiv a_{K}^{(\mu )+}a_{K}^{\left(\mu \right)}$. In summary, in the thermodynamic force space, the total entropy production strength behaves like a collection of K discretized independent one-dimensional harmonic oscillators, each with oscillation frequency $\omega_{K}$. It is important to stress that in the heuristic model introduced in Section 3.1, we assumed that the entropy production is proportional to the frequency in the space of thermodynamic forces. However, we know that such an assumption must be confirmed through the use of a rigorous and appropriate mathematical formalism. In this case, such a formalism is provided by the algorithm of the second quantization. This algorithm requires:

(1) Defining the canonically conjugated variables;
(2) Promoting such variables to operators;
(3) Providing the commutation rules;
(4) Defining the space where such operators act.

All this goes well beyond Equations (8) and (17) (for details, see [10]).

### 3.4. The Correspondence Principle in Relation to Einstein–Prigogine Fluctuation Theory

The contribution

(26) σ0=12∑μ=1n∑KkBωK

diverges. This expression represents the total entropy production in a macroscopic system arising from small fluctuations around thermodynamic equilibrium. To examine this term, we apply the Einstein–Prigogine theory of equilibrium fluctuations, which describes the probability *P* of observing a state where the values of $\alpha_{\mu }$ lie between $\alpha_{\mu }$ and $d\alpha_{\mu }$ are as follows:

(27) $$Pd\alpha_{1}\ldots d\alpha_{n}=P_{0}\exp\left(-\Delta_{I}S/k_{B}\right)d\alpha_{1}\ldots d\alpha_{n}$$

where $P_{0}$ normalizes to unity. Einstein’s fluctuation theory can be found in [38]. Here, the probability of finding a state where values of $\alpha_{i}$ lie between $\alpha_{i}$ and $\alpha_{i}+d\alpha_{i}$ involves the total entropy $dS_{Tot}=d_{I}S+d_{e}S$. Prigogine introduced a fundamental change in Einstein’s equation. In Prigogine’s fluctuation theory, only the entropy production contribution, $d_{I}S$ enters into the probability expression of the probability, not the entropy reversible contribution $d_{e}S$. As stated by Prigogine, this latter formulation is more general than Einstein’s formulation, which applies only to some particular transformations, such as adiabatic or isothermal changes [17] (see chapter 4, section 3, page 49). Notice that expression (27) applies solely to small, spontaneous fluctuations around thermodynamic equilibrium, not to systematic deviations from equilibrium. Prigogine demonstrated the following important result: regardless of the nature of the thermodynamic system (whether hydrodynamic, chemical, etc.), the average entropy production due to spontaneous equilibrium fluctuations is [17]

(28) $$\bar{\Delta_{I}S}=\int \ldots \int \Delta_{I}S\;Pd\alpha_{1}\ldots d\alpha_{n}=\frac{n}{2}k_{B}$$

where *n* represents the number of independent thermodynamic forces. This fundamental result shows that “*Each irreversible process contributes the same terms $k_{B}/2$ to the average of entropy due to* (equilibrium) *fluctuations*”. Equation (28) is derived using Prigogine’s fluctuation theory and not Einstein’s fluctuation theory. Next, we need to determine the eigenvalues of the operator $\Delta_{I}S$ using the canonical commutation relations. The detailed steps for deriving the expression of the $\Delta_{I}S$ operator are provided in [10]. We finally obtain the following:

(29) $$\Delta_{I}S=\frac{1}{4V}\int \left({\hat{q}}_{\mu \nu }X^{\mu }X^{\nu }+q^{\mu \nu }\alpha_{\mu }\alpha_{\nu }\right)\sqrt{L}\;d^{n}X$$

where ${\hat{q}}_{\mu \nu }$ denotes a definite positive matrix. By performing a linear coordinates transformation such that

(30) $${\alpha^{\prime }}_{\lambda }=A_{\lambda }^{\kappa }\alpha_{\kappa },\;\mathrm{with}\;\;A_{\nu }^{\mu }\;\mathrm{with}\;\;A_{\lambda }^{\alpha }q^{\lambda \kappa }A_{\kappa }^{\beta }=I^{\alpha \beta }$$

and defining a suitable new set of dimensionless variables, we obtain

(31) ΔIS=∑μ=1n∑KkB∣X˜Kμ∣2+∣α˜μ,K∣2

The introduction of the operators of creation and destruction $a_{K}^{(\mu )+}$ and $a_{K}^{\left(\mu \right)}$ and the number operator $n_{K}^{\left(\mu \right)}$ yield

(32) ΔIS=∑μ=1n∑KkBnK(μ)+n2∑KkB

where the canonical commutation relations are applied. Equation (32) aligns with the ground state derived from the Einstein–Prigogine fluctuation theory, establishing a *correspondence principle* by setting [10]:

(33) n2∑KkB≡n2kB

This leads to the following expression for the entropy production operator

(34) ΔIS=∑μ=1n∑KkBnK(μ)+n2kB

having the discrete eigenvalues:

(35) ΔIS=∑μ=1n∑KkBnK(μ)+n2kB≥0

The positivity of these eigenvalues shows the agreement with the second law of thermodynamics.

## 4. Modeling β for Quasi-Ideal Gases in Statistical Physics

Our goal is to uncover the physical origin of the constant β in the CCRs. We begin with a straightforward assumption: *the expression for β can be derived through a statistical model of nano-gases*. For this purpose, we employ a simple heuristic model for a nano-gas, based on the following assumptions:

**(1)**  The limiting case is defined by a molecular impact parameter *b* approximately twice the Bohr radius, i.e., $b=2r_{B}$.
**(2)**  We assume a spherical-molecule model where classical statistical physics holds, alongside the Heisenberg principle.

In our model, the system is treated as a quasi-ideal nano-gas, meaning that the interactions between particles are weak enough to be approximated by ideal gas behavior, but mesoscopic effects are still present. This assumption simplifies the analysis while still capturing the key mesoscopic phenomena. The use of a statistical model aligns with the mesoscopic nature of the system, where fluctuations and the discrete nature of the collision process in low-dimensional systems play a dominant role. The model is built on the idea that entropy at the mesoscopic level is quantized, meaning that changes in entropy occur in discrete steps. The uncertainty relations derived in our model establish a fundamental limit to how precisely certain pairs of thermodynamic variables (such as entropy production strength and time) can be known. The assumption of quantized entropy allows for a clear derivation of these uncertainty relations, which are expected to hold in mesoscopic systems.

### 4.1. Contextual Explanation of the Assumptions

**Assumption** **1.** *Let us analyze the first assumption. The impact parameter b in scattering processes refers to the perpendicular distance between a particle’s trajectory and the scattering potential’s center. It determines the strength of the interaction and the nature of the scattering process. The Bohr radius, $r_{B}$, defines a typical scale for the size of atoms or their interaction range in quantum mechanics. For nano-gas systems, which may involve quantum-sized particles or quantum confinement effects, using $r_{B}$ provides a natural scale. Doubling the Bohr radius ($b=2r_{B}$) is theoretically justified as this approximation accounts for interaction ranges that are slightly extended due to the statistical averaging over particle positions. The choice of $b=2r_{B}$ as a threshold for determining when quantum effects become significant is based on several factors. When the impact parameter b is much larger than the Bohr radius, $B\gg r_{B}$, the scattering process can often be treated classically. This is because the particle’s trajectory is far enough from the atomic center that quantum effects, such as wavefunction interference, become negligible. When b approaches the Bohr radius, the scattering interaction occurs at distances where quantum effects, such as the uncertainty in the particle’s position and momentum, become important. $b=2r_{B}$ is often used as an approximate threshold because it represents a critical distance where the quantum mechanical description starts to deviate significantly from the classical approximation. For distances $b<2r_{B}$, quantum mechanical effects dominate, and a wavefunction-based description is necessary. This approximation is particularly useful in mesoscopic systems, where the particles are neither fully macroscopic (classical) nor fully microscopic (quantum). At these scales, $b=2r_{B}$ offers a practical dividing line (see, for instance, [39,40,41]).*

#### Comparison with Other Models

Other potential models for determining when quantum effects become important in scattering processes might use different approximations or criteria based on more complex interactions or more precise quantum mechanical descriptions. One of some alternatives is provided by the *wavefunction overlap models* (for instance, ref. to [42]). These models focus on the overlap between the wavefunctions of the scattering particle and the target. Quantum effects become significant when the wavefunctions begin to overlap significantly, which typically occurs at distances on the order of $r_{B}$. This model provides a more detailed, but complex, description of the transition between classical and quantum behavior. Another model can be adopted when we consider the *Lennard-Jones potentials* (for instance, ref. to [43]). In molecular scattering, Lennard-Jones potentials describe the interaction between atoms or molecules, combining attractive and repulsive forces. These potentials suggest that quantum effects become important at distances where the potential energy is comparable to the kinetic energy of the particle, which may differ slightly from the simple approximation of $b=2r_{B}$. While the Lennard-Jones model provides a more accurate description of molecular interactions, it introduces additional complexity by requiring detailed knowledge of the interaction potential for each system. For charged particles, the *Coulomb interaction* dominates the scattering process (for instance, ref. to [44]). Quantum mechanical effects in Coulomb scattering become significant when the distance between particles is comparable to the de Broglie wavelength of the particle, rather than the Bohr radius. This approach would result in a different threshold for quantum effects but is specific to charged particle systems. Other models might use parameters derived from intermolecular potentials like *van der Waals interactions*, where *b* would be adjusted based on empirical data (for instance, ref. to [45]). The approximation of $b=2r_{B}$ might be simpler and more convenient, especially if detailed molecular interactions are not available or required for mesoscopic system modeling. Finally, semiclassical models, such as the WKB (*Wentzel-Kramers-Brillouin*) approximation, provide a more nuanced description of the transition between classical and quantum behavior (for instance, ref. to [46]). In these models, quantum effects are considered significant when the action of the system (in terms of Planck’s constant) becomes comparable to the characteristic scales of the problem.

Anyhow, the choice of $b=2r_{B}$ provides a simple and universal rule of thumb for determining when quantum effects should be considered. While more complex models exist that might involve other parameters, this approximation captures the essential physics with minimal complexity.

**Assumption** **2.** *The Bohr radius is about $r_{B}=5.29\times 10^{-11}$ m. The diameter of molecules in nanomaterial can vary significantly. Our calculation assumed a perfect sphere for the molecule of nano-gas (which is not always the case). So, while the actual ratio might depend on the specific nano-gas, a molecular radius of the order $r_{M}∼5\times 10^{-10}$ m and $r_{b}/r_{M}∼0.1$ is a reasonable estimate for the order of magnitude [47].*

## 5. Determination of Constant β and Comparison with the Experimental Value

**Figure 2:**
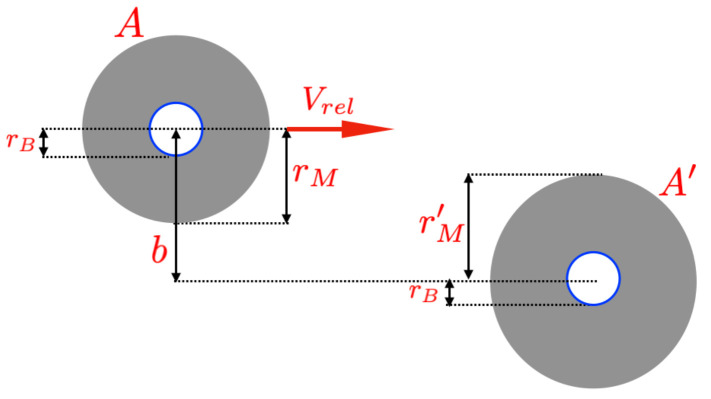
Collision of two molecules in a quasi-ideal nano-gas. The impact parameter *b* represents the distance between the centers of the two molecules. In our heuristic model, we consider the molecules to be identical and spherical. The model posits that when $b\leq 2r_{B}$, the two molecules collide and are influenced by the Heisenberg uncertainty principle. This limit is achieved when the distance between the molecules is $b=2r_{N}$, corresponding to the close packing fraction for spheres (i.e., $n=\pi /\left(3\sqrt{2}\right)V_{M}^{-1}$).

Figure 2 illustrates the classical spherical model used to depict molecules in nano-gases. In our heuristic model, the impact parameter *b* is set at twice the Bohr radius, and all molecules in the nano-gas are assumed to be identical (thus, $r_{M}^{\prime }=r_{M}$, where $r_{M}$ represents the molecular radius). The volume occupied by a molecule is given by $V_{M}=4/3\pi r_{M}^{3}$.

At thermal equilibrium, the classical uncertainty associated with measuring the entropy production strength σ is expressed as follows [48]:

(36) $$\Delta \sigma ∼\frac{\Delta E}{T\tau }$$

Here, τ represents the collision time, *T* is the system’s temperature, and ΔE denotes the energy uncertainty of the classical system. Equation (36) can be derived from the first thermodynamic law which, in the absence of work on or by the system, states that dE=TdS with dE and dS denoting the variation of the energy and the variation of the total entropy of the system, respectively. From Prigogine’s law, dS is given by two contributions: a piece due to reversible transformations and a piece due to dissipative processes [17]. In our case, dissipation arises only from collisions, and there is no reversible entropy flow during the collision process. So, $dS=d_{I}S$ with $d_{I}S$ denoting the variation of the entropy production of the system. The entropy production strength σ is given by $\sigma =d_{I}S/dt$. In scattering processes, if the temperature of the system remains constant and assuming that the smallest time steps are equal to the collision time τ, the variation of the entropy production strength reads Δσ∼ΔE/(Tτ). In classical statistical mechanics, the equipartition theorem connects the temperature of a system to its average energies as follows:

(37) $$\frac{1}{2}m_{M}\bar{v^{2}}=\frac{3}{2}k_{B}T$$

$\bar{v^{2}}$ and $m_{M}$ are the mean square speed and the molecule mass, respectively. The expression for the collision time is obtained from statistical theory [47]:

(38) $$\tau =\frac{1}{n\sigma_{cs}{\bar{V}}_{rel}}$$

where $\sigma_{cs}=\pi b^{2}=4\pi r_{B}^{2}$ is the cross-section. *n* and ${\bar{V}}_{rel}$ are the number of molecules for unit volume and the mean relative velocity, respectively. ${\bar{V}}_{rel}$ is linked to the root mean square speed by the relation $\bar{V_{rel}^{2}}=2\bar{v^{2}}$ [47]. If we do not make a distinction between the mean of the square and the square of the mean, we have ${\bar{V}}_{rel}≃\sqrt{2\bar{v^{2}}}$ [47]. By combining all these expressions we obtain

(39) $$\Delta t\Delta \sigma \;=\;\left(\Delta t\Delta E\right)\frac{3n\sigma_{cs}}{\sqrt{2}m_{M}\sqrt{\bar{v^{2}}}}k_{B}\;\geq \;\frac{\hbar }{2}\frac{3n\sigma_{cs}}{\sqrt{2}m_{M}\sqrt{\bar{v^{2}}}{|}_{Max.}}k_{B}$$

where the Heisenberg uncertainty principle

(40) $$\Delta t\Delta E\geq \frac{\hbar }{2}$$

has been taken into account, with *ℏ* denoting the reduced Planck constant (ℏ=h/(2π)). In this context, Δt and ΔE represent the uncertainties associated with time and energy measurements of the system, respectively. Based on our preliminary model, molecules collide with one another when the impact parameter reaches its minimum value, which occurs when

(41) $$n\sigma_{cs}=3\eta \frac{r_{B}^{2}}{r_{M}^{3}}$$

Here, η represents the packing fraction, while $r_{M}$ denotes the radius of the average molecule within the nanomaterial. Perfect packing is achieved when η=1.

### 5.1. Impact of Packing on Entropy Production

To conduct a sensitivity analysis for β, where β quantizes entropy production, we need to focus on how deviations from perfect packing influence β. In this setting, perfect packing could refer to an idealized arrangement of particles within nano-gas, where their spatial configurations are tightly constrained, leading to minimal entropy production per unit of fluctuation. Deviations from this perfect packing introduce disorder, which likely affects both the entropy production and the estimate of β. When η=1, particles are arranged optimally, minimizing the system’s entropy production. Let us assume that $\beta_{Ideal}$ is the value of β under perfect packing conditions. Imperfections in packing (e.g., due to thermal agitation or system size fluctuations) introduce randomness in particle positions. We can model this by introducing a packing efficiency factor, η, where η=1 represents perfect packing, and η<1 represents increasingly disordered configurations. The entropy production rate σ is influenced by the system’s disorder. If $\sigma_{Ideal}$ is the entropy production under perfect packing, deviations in packing increase disorder and therefore increase entropy production. Let us assume that entropy production depends on packing efficiency as follows:

(42) $$\sigma =\sigma_{Ideal}\left(1+f(\eta )\right)$$

where f(η) is a function that describes how much the entropy production increases with reduced packing efficiency. A simple linear model could be as follows:

(43) $$f\left(\eta \right)=\frac{1-\eta }{\eta }$$

So, as η decreases, the entropy production rate increases. In our work, β controls the quantization of entropy production. In real systems, perfect packing is nearly impossible due to thermal fluctuations, interactions, and other effects. As a result, the actual value of η is likely lower than η=1. By considering deviations from perfect packing, this model gives a more realistic estimate of η for practical applications. The main conclusion of our analysis is that the minimum value of the σ occurs in the ideal case when η=1. In this scenario, the packing is perfect, and the entropy production is at its minimum. The product $n\sigma_{cs}$ gives an estimate of the collision rate or how often molecules will encounter each other in a given volume. The larger this value, the more frequent the collisions. To minimize $n\sigma_{cs}$, *n* should be as large as possible, which occurs when the system is packed most efficiently (i.e., close packing). However, because the packing is very efficient, the free space available per molecule is minimized, which reduces the chance of collisions, despite the high number density. So, to minimize $n\sigma_{cs}$, we need to maximize the space available for the molecules to move while minimizing the likelihood of collisions. This happens when the number density *n* is maximized, which occurs in the close-packing scenario. In close packing, the molecules are tightly packed without voids, meaning their occupied volume is minimized. For spherical molecules with radius $r_{M}$, the volume of a single molecule is approximately $V_{M}=\frac{4}{3}\pi r_{M}^{3}$. The close packing fraction for spheres is known to be about $\eta =\pi /(3\sqrt{2})$ for an idealized close-packed structure (like FCC (face-centered cubic) or HCP (hexagonal closest packed) lattices) [49]. Thus, in this scenario, taking into account the effective occupied volume per molecule, the minimized $n\sigma_{cs}$ is as follows:

(44) $$n\sigma_{cs}=\frac{4\pi r_{B}^{2}}{V_{occupied}}=\frac{4\pi r_{B}^{2}}{V_{M}}\left(\frac{\pi }{3\sqrt{2}}\right)=\frac{\pi }{\sqrt{2}}\frac{r_{B}^{2}}{r_{M}^{3}}$$

### 5.2. Estimation of β

We can now estimate β approximately:

(45) $$\begin{array}{cc} \Delta t\Delta \sigma & \geq \frac{3\pi }{4}\frac{\hbar }{m_{e}cr_{B}}{\left(\frac{r_{B}}{r_{M}}\right)}^{3}\left(\frac{m_{e}}{m_{M}}\right)k_{B}\\ \; & =\frac{3\pi }{4}\alpha {\left(\frac{r_{B}}{r_{M}}\right)}^{3}\left(\frac{m_{e}}{m_{M}}\right)k_{B}=\beta k_{B} \end{array}$$

Here, $m_{e}$ represents the electron mass, c is the speed of light, and α denotes the fine-structure constant. While the electron mass appears in Equation (45), it does not affect the outcome, as the product $\alpha m_{e}$ is independent of the electron’s mass. However, highlighting this quantity is valuable for its physical meaning. From Equation (45) we find

(46) $$\beta =\frac{3\pi }{4}\alpha \left(\frac{V_{B}}{V_{M}}\right)\left(\frac{m_{e}}{m_{M}}\right)=\frac{3\pi }{4}\alpha \chi$$

where $V_{B}/V_{M}$ represents the ratio of the Bohr volume (characterizing atomic volume via the Bohr radius) to the volume of a single molecule, and $m_{e}/m_{M}$, is the mass ratio between an electron and a molecule in the material. We define $\chi \equiv \left(V_{B}m_{e}\right)/\left(V_{M}m_{M}\right)$ as the parameter *Bohr-Molecular Ratio* (BMR).

### 5.3. Discussion

As previously said, $r_{B}/r_{M}∼0.1$ is a reasonable estimate for the order of magnitude [47]. So, $V_{B}/V_{M}$ is of the order of $10^{-3}$. The mass of molecules in nanomaterials can vary significantly. However, the mass is likely several thousand to millions of times heavier than an electron for many common molecules. Therefore, saying the ratio between the electron mass and an average molecule’s mass is about the electron-to-proton mass ratio captures the significant difference in scale between these two quantities. To determine the ratio between the mass of an electron and an average molecule in a system on a mesoscopic level, we need to consider the mass of the entire molecule, which includes the masses of all the atoms it contains, along with their constituent protons, neutrons, and electrons. The mass of an electron is approximately $m_{e}=9.109\times 10^{-31}$ kilograms. A nanomaterial can be composed of a wide range of elements and have diverse molecular structures, so it is challenging to give a single specific ratio without knowing the exact composition of the nanomaterial. One example is graphene, a two-dimensional nanomaterial composed of a single layer of carbon atoms arranged in a hexagonal lattice. For our purposes, it is reasonable to assume that $m_{e}/m_{M}∼m_{e}/m_{p}$, with $m_{p}$ denoting the proton mass. Finally, the Bohr-Molecular Ratio $\chi =\left(V_{B}m_{e}\right)/\left(V_{M}m_{M}\right)$ is of the order of $10^{-6}-10^{-7}$. This suggests that the quantum effects described by the Bohr radius are not dominant on the mesoscopic scale and that classical physics can provide an adequate description of nano-gas behavior. Indeed,

(i) $V_{B}/V_{M}$ is proportional to the cube of the Bohr radius. At larger length scales, such as the mesoscopic level, the volume of interest ($V_{M}$) is significantly larger than the Bohr volume ($V_{B}$).
(ii) If the ratio $m_{e}/m_{M}$ is much smaller than unity, it suggests that the mass of the electron is negligible compared to the mass of the molecules. At the mesoscopic level, where we deal with large numbers of atoms and molecules, the mass of the molecules dominates, making the ratio $m_{e}/m_{M}$ very small.
(iii) The BMR $\chi =\left(V_{B}m_{e}\right)/\left(V_{M}m_{M}\right)$ compares the scale of fundamental atomic properties (Bohr radius and electron mass) to the scale of molecular properties (molecular volume and mass). The smallness of χ indicates the disparity in scales between the atomic level and the molecular level. So, the result that the discretization constant β is proportional to the ratio χ makes sense. This formulation effectively captures the disparity between atomic and molecular scales, providing a meaningful quantization of entropy production at the mesoscopic level. The use of fundamental constants and molecular properties supports the robustness of this result within the framework of mesoscopic thermodynamics.

By substituting the values α≃1/137, $V_{B}/V_{M}∼10^{-3}$, and $m_{e}/m_{M}∼m_{e}/m_{p}=0.5\times 10^{-3}$ into Equation (46) we obtain $\beta_{Theor.}∼0.9\times 10^{-8}$ which aligns with the experimental value found in [11]: $\beta_{Exp.}∼1.2\times 10^{-8}$.

### 5.4. Experimental Setups and Conditions for Determining β

*The Roldán E., et al. experiments*

In [11] the authors investigated entropy production in active fluctuations of hair-cell bundles, a biological system. To experimentally determine the parameter β, the authors employed a combination of techniques and statistical analysis:

(i) *Hair Cell Preparation*. Hair cells were isolated from bullfrog saccular epithelia and placed in a solution containing specific ions to maintain their physiological function.
(ii) *Optical Tweezers*. Hair bundles were trapped using optical tweezers, allowing for precise control and manipulation.
(iii) *High-Speed Video Microscopy*. The motion of the hair bundle was recorded using high-speed video microscopy, capturing the fluctuations at a high temporal resolution.

The recorded videos were analyzed to extract a time series of the hair bundle’s position. Statistical methods were successively used to analyze the time series data and estimate the probability distribution of the hair bundle’s position. Finally, the fluctuation theorem, a fundamental result in nonequilibrium statistical mechanics, relates the probability of observing a certain trajectory to the entropy production along that trajectory. By comparing the observed probability distribution with the theoretical prediction from the fluctuation theorem, the parameter β can be inferred. However, experimental noise can introduce uncertainties in the measurements, potentially affecting the accuracy of the estimated β. To mitigate this, in [11] the authors employed advanced signal processing techniques to reduce noise and improve the signal-to-noise ratio. The theoretical framework used to analyze the data relies on certain assumptions, such as the Markovian nature of the process and the validity of the linear response approximation. These assumptions may not be strictly valid in all cases, which could introduce errors in the estimation of β. To ensure the reliability and accuracy of their results, the experimental results were compared with theoretical predictions based on different models, such as Langevin equations and Fokker–Planck equations. The sensitivity of the estimated β to variations in experimental parameters, such as temperature and ionic concentration, was assessed. Finally, the results were compared with previous studies on similar systems to ensure consistency. By carefully considering these potential sources of error and implementing appropriate validation steps, the authors were able to obtain reliable estimates of the entropy production strength for the hair-cell bundle system.

*Experimental determination of β*

The experimental determination of β involves measuring entropy production and related thermodynamic variables in such systems under controlled conditions. The systems in question are typically at the mesoscopic scale—nanomaterials, nanoparticles, or nano-gases—where thermodynamic fluctuations are significant. In such systems, the entropy production is not continuous but exhibits quantized behavior, which can be observed experimentally through precise measurements of the system’s energy flux, particle movement, and heat exchange. The temperature of the system, along with its energy exchange with the surroundings, is measured in the experiments. This involves using calorimetry techniques at very small scales to track how much heat is generated or absorbed as the system moves toward equilibrium. Modern experiments use sophisticated techniques, like high-resolution microscopy, to track the motion of particles at the nanoscale. This motion helps determine the rate of entropy production and the fluctuations in the system. These setups involve systems driven out of equilibrium (e.g., by applying external forces or gradients). Entropy production in these conditions can be quantized, and β is derived by analyzing these quantized levels in comparison with the theoretical prediction. The experimental determination of β is performed by measuring the total entropy production in various types of mesoscopic systems and by looking for the conditions under which the entropy production becomes quantized. For instance:

(i) *Nanoparticle Systems*. Experiments involving nanoparticles suspended in a fluid or trapped using optical tweezers allow for precise control of particle dynamics. These dynamics, under thermal agitation, lead to entropy production that can be measured.
(ii) *Nano-gases*. In nano-gases, the behavior of particles can be studied by controlling their volume, temperature, and interaction forces. By studying the collective behavior of these particles, experimentalists can measure entropy production and compare it with the expression for β predicted by our model.

However, differences could arise from how well the experimental system conforms to the assumptions of the theoretical model (e.g., treating the system as a quasi-ideal nano-gas). Imperfections in the system, noise in measurements, and external influences might cause slight deviations from the predicted β. The size of the mesoscopic system could introduce corrections not accounted for in the theoretical model. Then, we have to introduce a quantitative framework for evaluating the uncertainties in measured and predicted quantities related to β and the entropy production strength. Uncertainty in measurement or prediction quantifies the range of values within which the true value likely lies. It can arise from various sources, including experimental errors, model limitations (due to the adopted simplified assumptions), and statistical fluctuations (i.e., the random variations in data due to sampling processes). Confidence intervals provide a range of values within which a population parameter (like β or the entropy production) is likely to lie with a certain level of confidence. Appropriate statistical tests, such as *t*-tests and ANOVA, can be used to compare theoretical and experimental β values using hypothesis tests (e.g., *t*-tests) to verify that differences are statistically insignificant within a specified confidence level. Additionally, if multiple datasets or experimental trials for β are available, a chi-squared test is useful to evaluate whether observed variations from predicted values fall within expected limits (for instance, refer to [50,51,52,53,54]). We can imagine an experiment that can measure β.

*Setup of the experiment*

The experiments can be carried out by using a quasi-ideal nano-gas, such as a confined cloud of noble gas atoms (e.g., helium or argon) in a mesoscopic trap. These gases exhibit minimal interatomic interactions, closely mimicking the theoretical assumptions for β. The confinement could be achieved with an optical or magnetic trap, creating a well-controlled environment where particles are isolated from external interference and operate at a mesoscopic scale. The nano-gas should be cooled to just above absolute zero, ideally within the milliKelvin range, to reduce thermal fluctuations. This allows thermal effects to emerge in the entropy measurements. We should ensure the trap dimensions are well-defined, ideally with boundaries at the scale of nanometers, to reflect the physical dimensions used in the model. Atomic force microscopy (AFM) or high-resolution electron microscopy (HREM) can measure and confirm the confinement area. Achieving and maintaining a stable low-temperature environment is critical, as thermal fluctuations can obscure β. Cryogenic cooling systems with feedback control could be utilized for precise temperature maintenance. Furthermore, the system should be isolated from electromagnetic interference and vibrations that could introduce errors in entropy measurements. Shielding and anti-vibration mounts for optical tables are recommended.

*The measurements process*

The measurement of entropy production may be performed by adopting the following procedure. The high-precision microcalorimeters to measure heat exchange may be used. This measure provides the integral to calculating entropy production in the system. Devices such as silicon-based nano-calorimeters, which offer high sensitivity, can measure minute heat changes in the system. Successively, we may apply laser spectroscopy to monitor particle dynamics within the trap. Laser-induced fluorescence or Raman spectroscopy would allow tracking of the position and energy levels of individual atoms, providing data on system fluctuations and entropy changes in real-time.

*Statistical analysis of the obtained results*

As mentioned above, the statistical analysis can be performed by using hypothesis testing (e.g., *t*-tests or ANOVA) to compare measured values of β across different density and temperature settings, ensuring statistical robustness. The chi-squared tests or goodness-of-fit analysis to validate whether observed values align with theoretical predictions must be applied successively. Finally, the confidence intervals for measured values should be established to assess consistency across trials.

### 5.5. Proposed Experiments and Simulations

Potential experimental application to validate the predictions reported in [10] and herein presented involves studying entropy production quantization in controlled nanoscale systems, such as nano-gases confined in optical traps or quantum dots. These systems allow precise control and measurement of thermodynamic variables, making it possible to observe entropy production strength as a function of system frequencies. Simulations could focus on stochastic models of these systems, using Langevin dynamics or Monte Carlo methods to compare predicted quantization with simulated results, particularly under varying thermodynamic forces and system sizes. Here are some concrete examples based on experiments and simulations to explore and validate the quantization of entropy production at the mesoscopic scale, as proposed in this paper and in [10].

*Nano-Gas Systems in Optical Traps*

Let us confine nano-gas (e.g., rarefied noble gases like argon or xenon) within an optical trap at low temperatures. By precisely controlling the trap’s frequency and measuring thermodynamic fluxes (heat and particle flows), the entropy production strength could be assessed and compared against quantized predictions. Optical traps allow for detailed observation of fluctuations in mesoscopic systems, making them ideal for studying the relationship between system frequencies and entropy production.

*Quantum Dot Systems*

Let us use semiconductor quantum dots as a platform to study energy transfer and dissipation in mesoscopic electronic systems. By varying excitation frequencies and monitoring heat dissipation and current noise, researchers can probe the quantization of entropy production. Quantum dots are tunable and exhibit strong quantum confinement effects, providing a highly controlled environment for testing thermodynamic predictions at the nanoscale.

*Molecular Motors or Active Matter*

Let us investigate entropy production in active matter systems such as artificial molecular motors or colloidal particles driven by external forces. Measure the energy input and dissipation rates across varying driving frequencies. These systems inherently operate far from equilibrium, and their dynamics are sensitive to the quantized nature of mesoscopic entropy production.

*Microfluidic Devices*

Let us create microfluidic setups where fluids are pushed through narrow channels under controlled pressure gradients. By measuring the energy dissipation and fluctuations in flow at various frequencies, entropy production rates can be correlated with system dynamics. Microfluidic systems mimic mesoscopic behaviors and offer precision control over thermodynamic variables.

*Stochastic Simulations of Langevin Dynamics*

Let us implement stochastic simulations (e.g., Langevin or Fokker–Planck equations) for particles under external forcing in mesoscopic systems. Introduce periodic driving forces to vary system frequencies and evaluate entropy production numerically. These simulations can directly test the quantization hypothesis by linking entropy production with discrete frequency modes.

These examples should offer feasible pathways for experimentalists and experts in computation to validate our theoretical predictions.

## 6. Perspective

The quantization model for entropy production at mesoscopic scales has broad implications for mesoscopic thermodynamics, as well as applications in fields such as nanotechnology, materials science, and possibly even quantum information theory. Here is a deeper look into these implications:

(i) *Advancement of mesoscopic thermodynamics*

The model’s derivation of a quantized constant β, suggests that even at mesoscopic scales—here classical and quantum descriptions typically overlap—thermodynamic behavior can be represented with discrete quantization. This bridges microscopic statistical mechanics and macroscopic thermodynamics, potentially paving the way for a consistent thermodynamic framework that extends seamlessly from nano to macro scales. Furthermore, by introducing a fundamental limit to fluctuations in mesoscopic systems, the quantized entropy production defines precision limits in measurements at nanoscales, impacting how experimental data in mesoscopic and nanoscale thermodynamics are analyzed.

(ii) *Impact on nanotechnology and nanoscale devices*

Understanding and quantifying entropy production at nanoscales is crucial for designing more efficient nanoscale devices, like sensors and actuators, where heat and energy dissipation are critical. The ability to predict or even control entropy production could lead to nanodevices that operate at higher efficiencies and lower energy costs. In nanoelectronics, entropy fluctuations often introduce instabilities. A quantized approach to entropy production might enable more predictable stability thresholds, allowing engineers to design devices that are resilient to fluctuations and have longer lifespans. For nano-lasers, where particle interactions and coherence play a significant role, the model provides a basis for determining the conditions under which these devices may operate reliably with minimal entropy production, potentially leading to innovations in photonics and optoelectronics.

(iii) *Material science and novel material design*

The quantized entropy parameter β may serve as a design criterion in developing materials that require precise control of thermodynamic properties, like thermal conductivity and electrical conductivity at the nanoscale. This is especially relevant for thermoelectric materials and metamaterials designed for heat management. Understanding entropy production quantization could impact the study of self-assembling nanomaterials. If entropy production is quantized, this could lead to predictable assembly patterns, enabling the creation of novel materials with tunable, complex structures based on controlled entropy-driven processes.

## 7. Conclusions

A core aim of the *Brussels School of Thermodynamics* was to explore mesoscopic systems to uncover the fundamental laws that govern them. Inspired by this objective and bolstered by recent experimental findings, we established canonical commutation rules (CCRs) applicable at the mesoscopic scale in [10]. These CCRs reveal that, as we approach the mesoscopic level, simultaneous measurements of canonically conjugate variables become increasingly uncertain. For instance, the uncertainties Δσ and Δt in the values of entropy production intensity and time are bound by ΔtΔσ≥ kB/2; thus, increasing precision in measuring one quantity reduces the accuracy in measuring the other. Our findings indicate that quantities like total entropy production can indeed be discretized at this scale. Additionally, the *ultraviolet divergence problem* is resolved by applying the *correspondence principle* to Einstein—Prigogine fluctuation theory in the macroscopic limit. Next, we address two essential questions: (1) *Is β a universal constant?* and (2) *Does β represent the absolute minimum?* To investigate, we analyzed a basic nano-gas model governed by the principles of classical statistical physics. In our model, the system is treated as a quasi-ideal nano-gas, meaning that the interactions between particles are weak enough to be approximated by ideal gas behavior, but mesoscopic effects, such as the emergence of the discrete nature of collisions and the role of thermodynamic fluctuations, are still present. This assumption simplifies the analysis while still capturing the key mesoscopic phenomena. The model assumes that the system is large enough to apply statistical mechanics but small enough that the nature of the collisions between particles is relevant. Our (heuristic) model suggests a ‘no’ to the first question, indicating that β is not a universal constant, while the answer to the second question is affirmative, implying that β represents the minimum possible value. Moreover, the theoretical value of β aligns closely with the experimental findings reported in [11]. However, while the reasoning presented addresses some relevant aspects, there are several limitations to consider. For instance, the assumption of the close-packing fraction for spheres ($\eta =\pi /(3\sqrt{2})$) may not always be feasible in real-world scenarios. Achieving perfect packing in nanomaterials may be challenging due to molecular size variations, steric hindrance, and thermal fluctuations. Real nanomaterials often exhibit imperfect packing, affecting their properties and behavior. Our approach centered on minimizing the average distance between molecules to reduce uncertainty in entropy production strength, thus simplifying the role of molecular interactions. In reality, however, both the nature and intensity of these interactions significantly influence the system’s entropy production and overall behavior.

Consequently, we conclude that Equation (46) supports the hypothesis that entropy production strength and thermodynamic variables conjugate to thermodynamic forces can indeed be discretized at the mesoscopic scale. In [55], the authors examined biomolecular systems - including molecular motors, transcription, and translation machinery, and other enzymatic processes - demonstrating that in a steady state, the dispersion of observables adheres to an uncertainty relation. Specifically, by studying a nonequilibrium chemical reaction catalyzed by an enzyme, where each step represents a completed enzymatic cycle, they showed that the product of (time-entropy production strength) is constrained by the following inequality:

(47) $$t\sigma \geq 2\frac{{\left(k^{+}-k^{-}\right)}^{2}\tau_{step}}{k^{+}+k^{-}}k_{B}=\beta k_{B}$$

where the steps to the right happen with a rate $k^{+}$, those to the left with a rate $k^{-}$, respectively, and $\tau_{step}$ is the elementary time step. The elementary time step in the context of a biased random walk for enzymatic reactions is typically considered to be the mean time it takes for the enzyme to complete one full cycle of reaction, encompassing all the steps mentioned above. Thus, we can state that the operators associated with the canonically conjugate variables do not commute with each other, and their commutators never vanish. Uncertainty relation has also been proposed in [56]. However, it is essential to highlight the key differences. In [56], the authors derive uncertainty relations tied to time, while in [10] it is explored a quantization principle that links the discretized entropy production to frequencies. In [10], the authors offer a connection between quantized entropy and observable mesoscopic behaviors, while [56] deals with general stochastic bounds.

Another noteworthy aspect is that the approach presented in [10] extends beyond systems governed by linear-response nonequilibrium. In this study, we limited ourselves to describing the quantization mechanism in the linear thermodynamic regime (the so-called Onsager region) where thermodynamic forces and conjugate fluxes exhibit a linear relationship. While the linear-response region serves as the foundational case, future work will explore the discretization of total entropy production, of variables conjugate to the thermodynamic forces, and of Glansdorff - Prigogine’s dissipative variable for systems operating outside of Onsager’s region. Using the field theory developed in [28,31,57,58], this expansion will incorporate not only quadratic terms but also cubic and higher-order contributions, such as terms of the form ${\left(a+a^{+}\right)}^{4}$. The updated expression for total entropy production will comprise two parts: the established result for Onsager systems and an additional *interaction term* non-commuting with the former—scaled by a coupling constant, ϵ. The discretized entropy production will then be derived through a perturbative expansion, assuming ϵ is sufficiently small. Practical examples of systems outside the Onsager region analyzed using this method are currently under investigation.

## Data Availability

Upon request, the author can provide the Mathematica software, Version 13.3 codes that implement the numerical calculations presented in this study.
